# 3,5-Dibromo-4-oxo-2,2,6,6-tetra­methyl­piperidin-1-yl oxide

**DOI:** 10.1107/S1600536811046812

**Published:** 2011-11-12

**Authors:** Guang-Zhou Zhu, Suo-Ping Xu, Cui-Yun Li

**Affiliations:** a Jiangsu Key Laboratory of Green Synthetic Chemistry for Functional Materials, Xuzhou Normal University, Xuzhou 221116, People’s Repulic of China; b State Key Laboratory of Pharmaceutical Biotechnology, Nanjing University, Nanjing 210093, People’s Republic of China

## Abstract

In the title compound, C_9_H_14_Br_2_NO_2_, the substituted ring exhibits a chair conformation. A crystallographic mirror plane, passing through the N atom, the O atoms and the C atom in the 4-position, bis­ects the mol­ecule.

## Related literature

For medical applications of similar compounds, see: Aubert *et al.* (2011 ▶); Brike (1990 ▶); Xu *et al.* (2009 ▶). For puckering parameters see: Cremer & Pople(1975 ▶).
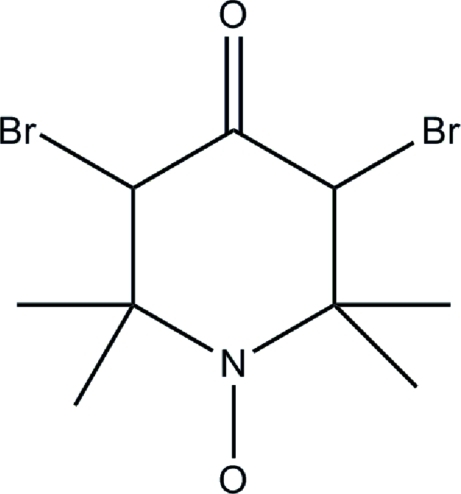


## Experimental

### 

#### Crystal data

C_9_H_14_Br_2_NO_2_
*M* *_r_* = 328.03
Orthorhombic, 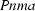

*a* = 11.6745 (9) Å
*b* = 16.0848 (14) Å
*c* = 5.9301 (4) Å
*V* = 1113.57 (15) Å^3^
*Z* = 4
Mo *K*α radiation
μ = 7.26 mm^−1^
*T* = 298 K
0.45 × 0.42 × 0.18 mm

#### Data collection

Bruker SMART CCD area-detector diffractometer
Absorption correction: multi-scan (*SADABS*; Bruker, 2000 ▶) *T*_min_ = 0.139, *T*_max_ = 0.355
5193 measured reflections
1018 independent reflections
774 reflections with *I* > 2σ(*I*)
*R*_int_ = 0.118

#### Refinement

*R*[*F* ^2^ > 2σ(*F* ^2^)] = 0.034
*wR*(*F* ^2^) = 0.089
*S* = 1.04
1018 reflections
72 parameters
H-atom parameters constrained
Δρ_max_ = 0.52 e Å^−3^
Δρ_min_ = −0.64 e Å^−3^

### 

Data collection: *SMART* (Bruker, 2000 ▶); cell refinement: *SAINT* (Bruker, 2000 ▶); data reduction: *SAINT*; program(s) used to solve structure: *SHELXS97* (Sheldrick, 2008 ▶); program(s) used to refine structure: *SHELXL97* (Sheldrick, 2008 ▶); molecular graphics: *SHELXTL* (Sheldrick, 2008 ▶); software used to prepare material for publication: *SHELXTL*.

## Supplementary Material

Crystal structure: contains datablock(s) global, I. DOI: 10.1107/S1600536811046812/bx2376sup1.cif

Structure factors: contains datablock(s) I. DOI: 10.1107/S1600536811046812/bx2376Isup2.hkl

Additional supplementary materials:  crystallographic information; 3D view; checkCIF report

## Figures and Tables

**Fig. 1.**
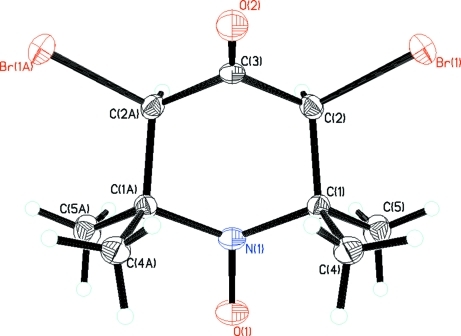
The molecular structure of (I) showing 30% probability displacement ellipsoids.

## supplementary crystallographic information

### Comment

3,5-dibromo-4-oxo-2,2,6,6-tetramethylpiperidin-1-yl oxide is an important intermediate medicament. It is synthesized in a wide range of medical applications (Aubert *et al.* (2011); Brike (1990); Xu *et al.* (2009)). The complete molecule of the title compound, C_9_H_14_Br_2_NO_2_, is generated by crystallographic mirror symmetry, with two O, one C in the 3-position and one N atom lying on the mirror plane, Fig1. The substituted cyclohexyl ring adopts a chair conformation ( Q_T_=0.562 (4)Å, θ =19.0 (4)°, φ =180.0 (12)° ), Cremer & Pople, (1975)

### Experimental

The title compound was synthetized by reaction between 4-Oxo-2,2,6,6-tetramethylpiperidin-1-yl oxide (2 mmol) and bromine (2 mmol), dissolved in CH_2_CH_2_Cl_2_ and mixed together for 2 h. Large block crystals were precipitated, filtered ,washed with ethanol and dried in air (yield 80%).

### Refinement

All H atoms were positioned geometrically(C—H = 0.96–0.98 Å,) and were refined as riding, with *U*_iso_(H) = 1.2*U*_eq_(carrier) or 1.5*U*_eq_(methyl C).

### Crystal data

**Table:**

C_9_H_14_Br_2_NO_2_	*D*_x_ = 1.957 Mg m^−^^3^
*M**_r_* = 328.03	Mo *K*α radiation, λ = 0.71073 Å
Orthorhombic, *P**n**m**a*	Cell parameters from 1704 reflections
*a* = 11.6745 (9) Å	θ = 3.5–26.6°
*b* = 16.0848 (14) Å	µ = 7.26 mm^−^^1^
*c* = 5.9301 (4) Å	*T* = 298 K
*V* = 1113.57 (15) Å^3^	Block, orange
*Z* = 4	0.45 × 0.42 × 0.18 mm
*F*(000) = 644	

### Data collection

**Table:**

Bruker SMART CCD area-detector diffractometer	1018 independent reflections
Radiation source: fine-focus sealed tube	774 reflections with *I* > 2σ(*I*)
graphite	*R*_int_ = 0.118
phi and ω scans	θ_max_ = 25.0°, θ_min_ = 3.5°
Absorption correction: multi-scan (*SADABS*; Bruker, 2000)	*h* = −13→12
*T*_min_ = 0.139, *T*_max_ = 0.355	*k* = −19→18
5193 measured reflections	*l* = −6→7

### Refinement

**Table:**

Refinement on *F*^2^	Primary atom site location: structure-invariant direct methods
Least-squares matrix: full	Secondary atom site location: difference Fourier map
*R*[*F*^2^ > 2σ(*F*^2^)] = 0.034	Hydrogen site location: inferred from neighbouring sites
*wR*(*F*^2^) = 0.089	H-atom parameters constrained
*S* = 1.04	*w* = 1/[σ^2^(*F*_o_^2^) + (0.0364*P*)^2^] where *P* = (*F*_o_^2^ + 2*F*_c_^2^)/3
1018 reflections	(Δ/σ)_max_ < 0.001
72 parameters	Δρ_max_ = 0.52 e Å^−^^3^
0 restraints	Δρ_min_ = −0.64 e Å^−^^3^

### Special details

**Table:**

Geometry. All e.s.d.'s (except the e.s.d. in the dihedral angle between two l.s. planes) are estimated using the full covariance matrix. The cell e.s.d.'s are taken into account individually in the estimation of e.s.d.'s in distances, angles and torsion angles; correlations between e.s.d.'s in cell parameters are only used when they are defined by crystal symmetry. An approximate (isotropic) treatment of cell e.s.d.'s is used for estimating e.s.d.'s involving l.s. planes.
Refinement. Refinement of *F*^2^ against ALL reflections. The weighted *R*-factor *wR* and goodness of fit *S* are based on *F*^2^, conventional *R*-factors *R* are based on *F*, with *F* set to zero for negative *F*^2^. The threshold expression of *F*^2^ > σ(*F*^2^) is used only for calculating *R*-factors(gt) *etc*. and is not relevant to the choice of reflections for refinement. *R*-factors based on *F*^2^ are statistically about twice as large as those based on *F*, and *R*- factors based on ALL data will be even larger.

### Fractional atomic coordinates and isotropic or equivalent isotropic displacement parameters (Å^2^)

**Table:**

	*x*	*y*	*z*	*U*_iso_*/*U*_eq_	
Br1	0.28737 (4)	0.42632 (3)	0.59566 (7)	0.0414 (2)	
N1	0.4892 (4)	0.2500	0.2492 (7)	0.0268 (11)	
O1	0.5770 (4)	0.2500	0.1217 (6)	0.0448 (11)	
O2	0.2994 (3)	0.2500	0.7814 (7)	0.0384 (10)	
C1	0.4591 (3)	0.3333 (2)	0.3498 (6)	0.0237 (9)	
C2	0.3343 (3)	0.3265 (2)	0.4364 (6)	0.0263 (9)	
H2	0.2844	0.3206	0.3045	0.032*	
C3	0.3177 (4)	0.2500	0.5827 (10)	0.0277 (13)	
C4	0.5446 (3)	0.3542 (3)	0.5367 (7)	0.0354 (10)	
H4A	0.5310	0.3188	0.6643	0.053*	
H4B	0.5354	0.4112	0.5808	0.053*	
H4C	0.6212	0.3456	0.4824	0.053*	
C5	0.4652 (4)	0.3975 (3)	0.1615 (8)	0.0418 (11)	
H5A	0.5425	0.4012	0.1069	0.063*	
H5B	0.4416	0.4507	0.2182	0.063*	
H5C	0.4155	0.3811	0.0405	0.063*	

### Atomic displacement parameters (Å^2^)

**Table:**

	*U*^11^	*U*^22^	*U*^33^	*U*^12^	*U*^13^	*U*^23^
Br1	0.0384 (3)	0.0281 (3)	0.0576 (4)	0.00706 (19)	0.00867 (18)	−0.0047 (2)
N1	0.024 (2)	0.033 (3)	0.023 (3)	0.000	0.0032 (17)	0.000
O1	0.039 (3)	0.050 (3)	0.046 (3)	0.000	0.0234 (19)	0.000
O2	0.048 (3)	0.034 (2)	0.033 (3)	0.000	0.0153 (19)	0.000
C1	0.025 (2)	0.022 (2)	0.024 (2)	−0.0017 (17)	0.0009 (14)	−0.0011 (16)
C2	0.023 (2)	0.024 (2)	0.033 (2)	0.0032 (17)	−0.0028 (14)	−0.0031 (17)
C3	0.011 (3)	0.026 (3)	0.046 (4)	0.000	−0.001 (2)	0.000
C4	0.024 (2)	0.034 (2)	0.047 (3)	−0.001 (2)	−0.0046 (17)	−0.007 (2)
C5	0.053 (3)	0.034 (2)	0.039 (3)	−0.001 (2)	0.006 (2)	0.007 (2)

### Geometric parameters (Å, °)

**Table:**

Br1—C2	1.942 (4)	C2—H2	0.9800
N1—O1	1.274 (5)	C3—C2^i^	1.518 (5)
N1—C1^i^	1.509 (4)	C4—H4A	0.9600
N1—C1	1.509 (4)	C4—H4B	0.9600
O2—C3	1.198 (6)	C4—H4C	0.9600
C1—C5	1.523 (6)	C5—H5A	0.9600
C1—C4	1.529 (5)	C5—H5B	0.9600
C1—C2	1.548 (5)	C5—H5C	0.9600
C2—C3	1.518 (5)		
			
O1—N1—C1^i^	115.0 (2)	O2—C3—C2^i^	125.8 (2)
O1—N1—C1	115.0 (2)	O2—C3—C2	125.8 (2)
C1^i^—N1—C1	125.3 (4)	C2^i^—C3—C2	108.3 (5)
N1—C1—C5	107.5 (3)	C1—C4—H4A	109.5
N1—C1—C4	109.2 (3)	C1—C4—H4B	109.5
C5—C1—C4	110.6 (3)	H4A—C4—H4B	109.5
N1—C1—C2	106.7 (3)	C1—C4—H4C	109.5
C5—C1—C2	109.6 (3)	H4A—C4—H4C	109.5
C4—C1—C2	112.9 (3)	H4B—C4—H4C	109.5
C3—C2—C1	111.5 (3)	C1—C5—H5A	109.5
C3—C2—Br1	110.9 (3)	C1—C5—H5B	109.5
C1—C2—Br1	111.6 (3)	H5A—C5—H5B	109.5
C3—C2—H2	107.5	C1—C5—H5C	109.5
C1—C2—H2	107.5	H5A—C5—H5C	109.5
Br1—C2—H2	107.5	H5B—C5—H5C	109.5
			
O1—N1—C1—C5	46.0 (5)	C4—C1—C2—C3	−69.8 (4)
C1^i^—N1—C1—C5	−159.6 (3)	N1—C1—C2—Br1	174.9 (2)
O1—N1—C1—C4	−74.1 (4)	C5—C1—C2—Br1	−69.0 (3)
C1^i^—N1—C1—C4	80.3 (5)	C4—C1—C2—Br1	54.8 (4)
O1—N1—C1—C2	163.5 (4)	C1—C2—C3—O2	111.8 (5)
C1^i^—N1—C1—C2	−42.1 (6)	Br1—C2—C3—O2	−13.2 (6)
N1—C1—C2—C3	50.2 (4)	C1—C2—C3—C2^i^	−65.4 (5)
C5—C1—C2—C3	166.4 (3)	Br1—C2—C3—C2^i^	169.5 (2)

Symmetry codes: (i) *x*, −*y*+1/2, *z*.
